# PCSK9 genetic variants, carotid atherosclerosis and vascular remodelling

**DOI:** 10.1136/openhrt-2025-003348

**Published:** 2025-10-10

**Authors:** Daniela Coggi, Joey Ward, Chiara Macchi, Bruna Gigante, Mauro Amato, Donald M Lyall, Beatrice Frigerio, Alessio Ravani, Daniela Sansaro, Nicola Ferri, Maria Giovanna Lupo, Massimiliano Ruscica, Fabrizio Veglia, Nicolo Capra, Antonio Gallo, Matteo Pirro, Kai Savonen, Douwe J Mulder, Roberta Baetta, Elena Tremoli, Jill P. Pell, Paul Welsh, Naveed Sattar, Damiano Baldassarre, Rona J Strawbridge

**Affiliations:** 1 Centro Cardiologico Monzino, Istituto di Ricovero e Cura a Carattere Scientifico, Milan, Lombardy, Italy; 2 Institute of Health and Wellbeing, University of Glasgow, Glasgow, UK; 3 Dipartimento di Scienze Farmacologiche e Biomolecolari, Università degli Studi di Milano, Milan, Italy; 4 Cardiovascular Epidemiology, IMM, Karolinska Institutet, Stockholm, Sweden; 5 Centro Cardiologico Monzino, Milano, Italy; 6 University of Glasgow, Glasgow, UK; 7 Dipartimento di Medicina, Università degli Studi di Padova, Padua, Italy; 8 Università degli Studi di Milano, Milano, Italy; 9 Maria Cecilia Hospital, Cotignola, Italy; 10 IHU-ICAN, Paris, France; 11 Department of Nutrition, Hôpital Pitié-Salpêtrière, Paris, France; 12 Internal Medicine Department of Medicine and Surgery, University of Perugia, Perugia, Italy; 13 Department of Clinical Physiology and Nuclear Medicine, University of Eastern Finland, Kuopio, Finland; 14 Institute of Public Health and Clinical Nutrition, University of Eastern Finland, Kuopio, Finland; 15 Department of Internal Medicine, University of Groningen, Groningen, Netherlands; 16 Section of Public Health and Health Policy, University of Glasgow, Glasgow, UK; 17 BHF GCRC, University of Glasgow, Glasgow, UK; 18 British Heart Foundation Glasgow Cardiovascular Research Centre, Institute of Cardiovascular and Metabolic Health, University of Glasgow, Glasgow, UK; 19 Department of Medical Biotechnology and Translational Medicine, Università degli Studi di Milano, Milan, Italy; 20 Department of Medicine Solna, Karolinska Institutet, Stockholm, Sweden

**Keywords:** Atherosclerosis, Carotid Artery Diseases, Genetic Association Studies, Biomarkers, Echocardiography

## Abstract

**Background:**

Circulating proprotein convertase subtilisin/kexin type 9 (PCSK9) is a crucial regulator of cholesterol metabolism. Loss-of-function variants in PCSK9 are associated with lower levels of circulating low-density lipoprotein cholesterol (LDL-C) and reduced cardiovascular disease (CVD) risk, while gain-of-function variants correlate with elevated LDL-C concentrations and increased CVD risk. This study investigated whether genetically determined LDL-C levels, proxied by four PCSK9 genetic variants, influence common carotid artery atherosclerosis.

**Methods:**

The analysis included 3040 European participants (mean age 64.2±5.4 years; 45.8% men) at high cardiovascular risk from the IMPROVE Study, alongside 49 088 individuals of white British ancestry (mean age 55.2±7.6 years; 47.9% men) from the UK Biobank (UKB). Ultrasonographic measurements of common carotid intima-media thickness (CC-IMTmean, CC-IMTmax, CC-IMTmean-max) were obtained. Four lipid-level affecting genetic variants in the *PCSK9* locus were selected for analysis, both individually and in a standardised Lipid-Lowering Allelic Score (LLAS), to assess their effects on LDL-C and PCSK9 levels in the IMPROVE cohort and on ultrasonographic measures in both IMPROVE and UKB.

**Results:**

In the IMPROVE cohort, *PCSK9* variants (rs11206510, rs2479409, rs11591147, rs11583680) exhibited expected effect directions, although not all statistically significant, on LDL-C and PCSK9 levels. The LLAS was negatively correlated with CC-IMTmean, CC-IMTmax and CC-IMTmean-max among women in IMPROVE, and among men and overall in UKB (all p<0.05). Effect sizes were comparable between cohorts.

**Conclusions:**

Genetic variants in the *PCSK9* locus influence LDL-C levels and CC-IMT, in keeping with proven benefits of PCSK9 inhibitors on atherosclerotic cardiovascular events.

**Key messages**

**WHAT IS ALREADY KNOWN ON THIS TOPIC**

- Proprotein convertase subtilisin/kexin type 9 (PCSK9) is an important regulator of cholesterol metabolism.
- Genetic variants in the *PCSK9* gene influence circulating levels of low-density lipoprotein cholesterol (LDL-C), with knock-on effects on risk of cardiovascular disease (CVD).

**WHAT THIS STUDY ADDS**

- This study assessed the impact of *PCSK9* genetic variants, associated with reduced circulating LDL-C levels, on atherosclerosis (measured by common carotid intima-media thickness (CC-IMT)).
- *PCSK9* genetic variants, individually or combined in a Lipid-Lowering Allelic Score (LLAS), demonstrated association with reduced CC-IMT in two European ancestry data sets.
- No robust sex differences in associations between the LLAS and CC-IMT were observed.

**HOW THIS STUDY MIGHT AFFECT RESEARCH, PRACTICE OR POLICY**

- These findings suggest that PCSK9 influences CVD via LDL-C levels, but the effect was modest and did not differ significantly by sex or use of lipid-lowering medication.

## Introduction

 Intima-media thickness (IMT) of extracranial carotid arteries, measured non-invasively using B-mode ultrasound, is an imaging measure of arterial wall thickness often used as a surrogate marker of clinical^1^ and subclinical atherosclerosis.^2^ IMT is directly associated with all traditional cardiovascular risk factors, such as hypercholesterolaemia,^3^ age,^3^ blood pressure^4^ and cigarette smoking,^5^ as well as with the prevalent^6^ and incident^6^ vascular events. More recently, some,^7 8^ but not all,^9 10^ studies demonstrated that circulating levels of proprotein convertase subtilisin/kexin type 9 (PCSK9) are also associated with carotid IMT. PCSK9 (encoded by the *PCSK9* gene) is a serine protease mainly synthesised and secreted by the liver that plays a role not only in atherosclerosis,^11^ but also in the development of cardiovascular diseases (CVDs).^12^

PCSK9 post-transcriptionally causes the degradation of the low-density lipoprotein receptor (LDLR)^13^ and thus indirectly increases plasma low-density lipoprotein cholesterol (LDL-C) levels.^13^ Since 2003, many *PCSK9* genetic variants with ‘gain-of-function’ (GOF)^12^ or ‘loss-of-function’ (LOF)^12^ effects have been described. In particular, GOF variants were consistently associated with higher LDL-C levels^12^ through reduced LDLR levels, resulting in a higher risk of CVD^12^ or cerebrovascular diseases,^14^ with the opposite effect being seen with LOF variants. While many studies have evaluated the relationship between *PCSK9* variants and CVD or cerebrovascular diseases, to our knowledge few studies have evaluated the relationship between *PCSK9* variants and subclinical atherosclerosis, with only variants E670G (rs505151), I474V (rs562556),^15^ Y142X (rs67608943), C679X (rs28362286)^16^ and R46L (rs11591147) being investigated.^16 17^

Here we investigated the impact of four *PCSK9* genetic variants known to influence LDL-C levels,^18^ individually and combined in a Lipid-Lowering Allelic Score (LLAS), on carotid IMT measures in a high CVD-risk cohort (IMPROVE) and a large general population cohort (UK Biobank (UKB)).

## Methods

### Selection of PCSK9 variants

The selection of four LDL-C-lowering germline genetic variants in the *PCSK9* locus was based on the Schmidt *et al*^18^ definition (described in the online supplemental material), and included: rs11206510 (build 37, chromosome 1:55505668), rs2479409 (1:55504650), rs11591147 (ie, R46L, 1:55505647) and rs11583680 (1:55505668). These four chosen variants fulfilled stringent criteria (described in the online supplemental material) and were robustly testable in both cohorts.

### Study cohorts and phenotyping

The IMPROVE Study design, eligibility criteria, aims and baseline evaluation have already been reported in detail.^19^ Briefly, a total of 3711 subjects (age range 54‒79 years), defined as high CVD risk based on the presence of at least three traditional vascular risk factors (eg, hypercholesterolaemia, hypertension, diabetes) but free from CVD and cerebrovascular diseases, were consecutively recruited in seven centres across five European countries, that is, Finland (Kuopio, two centres), Sweden (Stockholm), the Netherlands (Groningen), France (Paris) and Italy (Milan and Perugia) between 2004 and 2005. In 2023, the Institute of Public Health and Clinical Nutrition at the University of Eastern Finland in Kuopio revoked data usage permission for participants recruited to this centre. As a result, this study included the 3040 participants recruited from the remaining six centres.

During the baseline visit, participants completed a medical history, medication and lifestyle questionnaire, carotid ultrasound examination and blood sampling for standard biochemical tests and genotyping. Details of lipid measurements and carotid ultrasound examination have been previously published^19^ and are summarised in the online supplemental material. For this study, three indices of the common carotid arterial wall damage (CC-IMTmean, CC-IMTmax and CC-IMTmean-max) were used, as these were deemed most comparable to the measurements conducted by UKB. Circulating PCSK9 levels were measured using commercial ELISA kits (R&D Systems, Minnesota, USA) able to recognise free and LDLR-bound PCSK9^20^ (online supplemental material). Lipid-lowering medication included statins, fibrates, fish oil and/or resin.^19^

The IMPROVE Study complies with the rules of Good Clinical Practice and with the ethical principles established in the Declaration of Helsinki. Details of the six ethics committees are provided in the online supplemental material. All patients provided informed consent twice; once for general participation in the study and once for genotyping.

The UKB cohort has also been described previously.^21^ In short, the UKB included over 500 000 participants (age range 40–69 years at baseline) recruited from the general population who attended 1 of 22 centres across the UK between 2006 and 2010. A self-completed baseline questionnaire provided information on personal and family medical history, medication, and lifestyle, and blood samples were taken for standard biochemical assays and genotyping. A subset of individuals was invited to participate in a follow-up imaging visit (4–8 years after baseline), which included carotid ultrasound examination.^22 23^ For this study, only 49 088 unrelated self-reported white British ancestry individuals with CC-IMT ultrasonographic variable measures were included. Details of the carotid ultrasound examination have been published previously^24^ and are summarised in the online supplemental material. Participants were asked, ‘Do you regularly take any of the following medications’ where options included medication for cholesterol. Medication for cholesterol was considered to be lipid-lowering medication.

This study was conducted under project #71 392 (Principle Investigator RJS).

### Genetic data

IMPROVE subjects were genotyped using both Illumina Cardio-Metabo 200 k^25^ and Immunochip 200 k^26^ array platforms. Standard quality control was conducted on the individual chips as well as the combined data set, including: exclusion of samples with cryptic relatedness, low call rate (<95%) or ambiguous sex and exclusion of variants for low call rate (<95%), failing Hardy-Weinberg Equilibrium (p<1×10^-5^) or low call rate (minor allele frequency (MAF) >1%). Multidimensional scaling (MDS) components 1, 2 and 3 were calculated in PLINK^27^ to enable adjustment for population structure.

UKB participants were genotyped using either the Affymetrix BiLEVE Axion or Affymetrix UKB Axion array and imputed to 1000 Genomes, UK10K and Haplotype Reference Consortium (March 2018 release) reference panels.^28^ Standard genetic quality control (consistent with that described above), preimputation and postimputation, was conducted centrally by UKB. Genetic principal components were also computed by the central UKB team. Eight principal genetic components (PGC1–8) were used to enable adjustment for population structure. Only unrelated individuals of self-reported white British ancestry were included in the analyses.

The MAF and Hardy-Weinberg equilibrium *p* of variants in IMPROVE and UKB are shown in online supplemental table S1. The linkage disequilibrium (LD) between the variants in IMPROVE and UKB is demonstrated in online supplemental figure S1 (panels A and B, respectively). Of note, the Block indication is an artefact of the software and is not accurate to the LD measure used.

**Figure 1:**
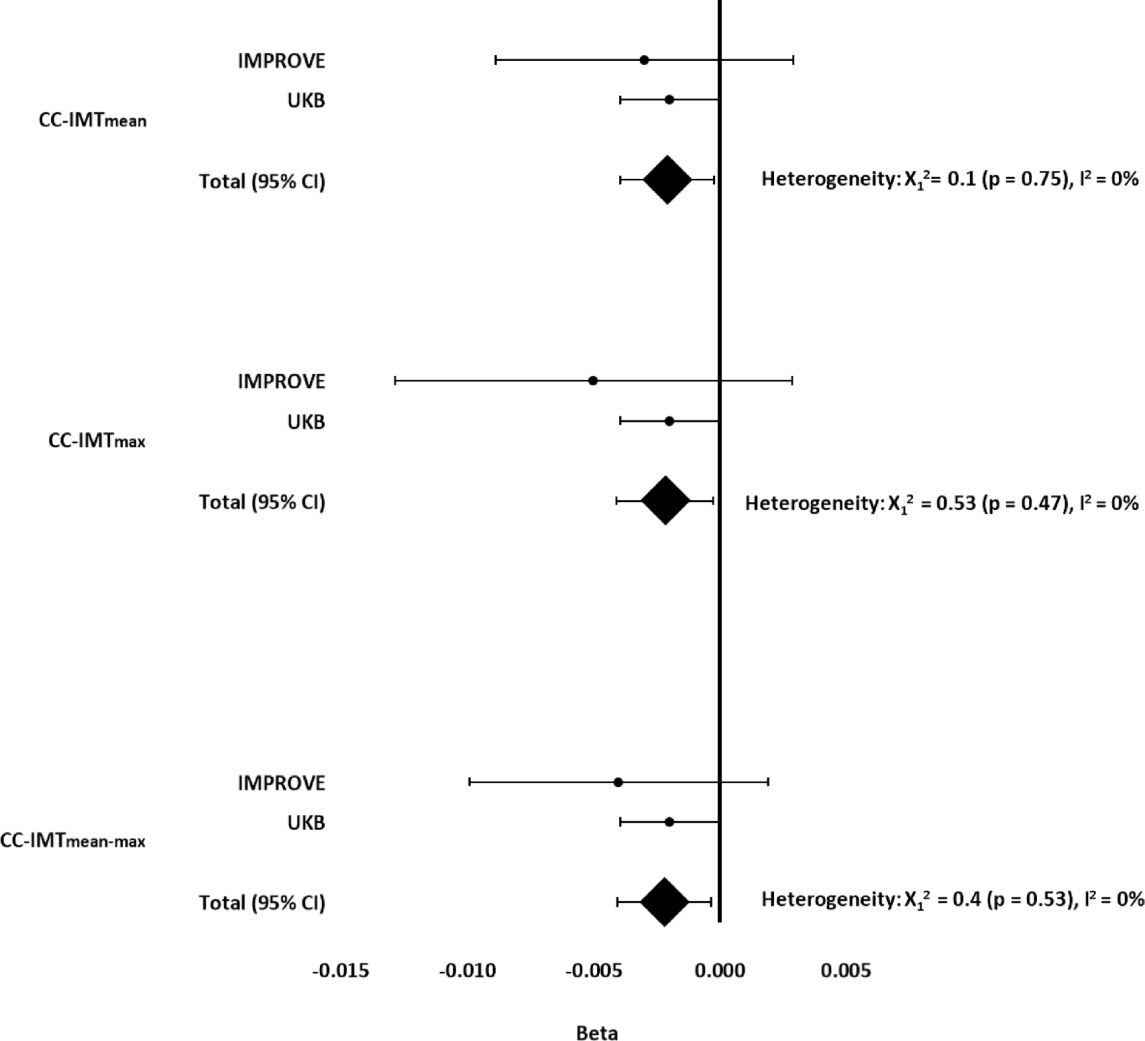
Individual betas (IMPROVE and UKB) and summarise beta (95% CI) of LLAS on CC-IMTmean, CC-IMTmax and CC-IMTmean-max. Beta adjusted for sex, age, population structure (MDS1–3) and use of lipid-lowering medication (statins, fibrates, fish oil and/or resin) in IMPROVE. Beta adjusted for sex, age, population structure (PGC1–8), genotyping chip and lipid-lowering medication in UKB. Values of p<0.05 were considered statistically significant. CC-IMTmean, average of means of intima-media thickness in the left and right common carotid arteries; CC-IMTmax, the highest value of maximum intima-media thickness in the left and right common carotid arteries; CC-IMTmean-max, mean of maximum intima-media thickness in the left and right common carotid arteries. LLAS, Lipid-Lowering Allelic Score; MDS1–3, multidimensional scaling components 1, 2 and 3; PGC1–8, eight principal genetic components; UKB, UK Biobank.

### Power calculations

For power calculations for individual variant analyses, we used a conservative estimate of variance explained for a model including age, sex and population structure of 0.001 (based on previous evidence of genetic effect sizes on CC-IMT^24^), which meant IMPROVE had 40% power to detect an effect, while UKB had >99% power.

### Statistical analyses

All continuous phenotypes were evaluated for normality. Triglycerides, PCSK9 and CC-IMT ultrasonographic variables were log normalised prior to analyses. Analyses of individual genetic variants were conducted using linear regression in PLINK, assuming an additive genetic model.

An unweighted LLAS was constructed by summing the number of LDL-C-lowering alleles (0, 1 and 2) of each variant and was used to assess the combined effect of the four variants. Only individuals with complete genotyping were included in LLAS analyses. The LLAS was standardised and analyses were conducted using general linear models (GLMs) in SAS V.9.4 (SAS Institute, Cary, North Carolina, USA) for IMPROVE and STATA (STATA Corp) for UKB.

In the IMPROVE Study, we assessed whether associations were observed between individual variants and lipids and PCSK9. Here, p<0.0125 was considered statistically significant (Bonferroni correction for four comparisons). Subsequently, we investigated the impact of individual variants on CC-IMTmean, CC-IMTmax and CC-IMTmean-max, considering p<0.0125 as statistically significant. Finally, the LLAS was tested for associations with CC-IMTmean, CC-IMTmax and CC-IMTmean-max. Here p<0.05 was considered statistically significant. Investigating the associations between individual variants and PCSK9, three models were considered: Model 1, adjusted for sex, age and population structure (ie, MDS1–3); Model 2, as model 1 plus use of lipid-lowering medication (statins, fibrates, fish oil and/or resin); Model 3, as model 1 plus independent determinants of PCSK9 (use of statins and/or fibrates, total cholesterol, high-density lipoprotein cholesterol, uric acid, personal history of hypertriglyceridaemia and/or hypercholesterolaemia, hip circumference, consumption of fish and/or wine, pack-years, family history of diabetes).^20^ Sensitivity analyses were also performed, stratified by sex. Individual variants were also assessed for effects on lipid levels adjusting for Model 2 in the whole group, in men and in women.

The effects on CC-IMT ultrasonographic variables, using Model 2, were tested by considering both the individual variants and LLAS. Sensitivity analyses were also performed, stratified either by sex or by lipid-lowering treatment. A GLM was used to adjust for confounding factors (Model 2) and to evaluate whether (1) LLAS was associated with PCSK9 levels after stratification by sex or by use of lipid-lowering medication (p<0.05 as statistically significant); (2) LLAS affected PCSK9 levels by interacting with sex or use of lipid-lowering medication (p<0.05 as statistically significant). Additionally, it was employed to estimate adjusted geometric means for PCSK9 levels by accounting for age and MDS1–3, across each combination of stratification factor values (women/men or treated/untreated) and LLAS.

In UKB, individual variants (p<0.0125 as statistically significant) and LLAS (p<0.05 as statistically significant) were tested for association with CC-IMTmean, CC-IMTmax and CC-IMTmean-max, adjusting for age, sex, genotyping chip, population structure (ie, PGC1–8) and use of lipid-lowering medication. Again, sensitivity testing included stratification by sex or by use of lipid-lowering medication, and the interaction (p<0.05 as statistically significant) between LLAS and sex or between LLAS and use of lipid-lowering medication was tested.

To assess whether the very different sample sizes between IMPROVE and UKB might have influenced the results, the estimated beta values of the relationships between LLAS and CC-IMT ultrasonographic variables from both studies were also analysed with a fixed-effects meta-analysis and visualised in a forest plot. Heterogeneity was assessed using the Q-test and the I^2^ statistic. p<0.05 was considered statistically significant.

### Patient and public involvement

There was no patient or public involvement in the design of this study.

## Results

The demographic features of IMPROVE and UKB participants are presented in table 1. Men and women were well represented in both studies. Subjects included in the IMPROVE Study were about 10 years older than participants of the UKB Study. IMPROVE is a high CVD-risk cohort, thus the higher values of CC-IMTmean, CC-IMTmax and CC-IMTmean-max observed in IMPROVE compared with the general population UKB are unsurprising, despite the more frequent use of lipid-lowering medication in IMPROVE compared with UKB (49.4% vs 12.0%, respectively).

**Table 1:** Baseline characteristics of the IMPROVE and UKB study cohorts

		IMPROVE			UKB	
	Men	Women	All	Men	Women	All
N (%)	1393 (45.8)	1647 (54.2)	3040	23 506 (47.9)	25 582 (52.1)	49 088
Age, years	64.0±5.3	64.4±5.5	64.2±5.4	55.9±7.7	54.9±7.7	55.2±7.6
BMI, kg/m^2^	27.5±3.7	26.9±4.7	27.2±4.3	27.1±3.7	26.0±4.5	26.5±4.2
Waist/hip ratio	0.97±0.07	0.87±0.07	0.92±0.09	0.92±0.06	0.80±0.07	0.86±0.09
LDL-C, mmol/L	3.43±0.94	3.75±1.05	3.61±1.01	na	na	na
HDL-C, mmol/L	1.12±0.30	1.37±0.37	1.26±0.36	na	na	na
Triglycerides, mmol/L	1.45 (1.02 to 2.11)	1.27 (0.92 to 1.81)	1.34 (0.96 to 1.95)	na	na	na
PCSK9, ng/mL	288 (227 to 358)	326 (266 to 395)	307 (247 to 381)	na	na	na
Lipid-lowering medication, n (%)	676 (48.7)	822 (49.9)	1498 (49.4)	3971 (17.2)	1801 (7.2)	5772 (12.0)
CC-IMTmean, mm	0.75 (0.67 to 0.85)	0.71 (0.65 to 0.78)	0.73 (0.66 to 0.81)	0.70 (0.61 to 0.80)	0.65 (0.59 to 0.73)	0.67 (0.60 to 0.76)
CC-IMTmax, mm	1.16 (1.02 to 1.45)	1.06 (0.97 to 1.21)	1.10 (0.98 to 1.31)	0.92 (0.81 to 1.08)	0.85 (0.77 to 1.00)	0.89 (0.77 to 1.04)
CC-IMTmean-max, mm	1.05 (0.94 to 1.21)	0.98 (0.91 to 1.10)	1.01 (0.92 to 1.14)	0.81 (0.71 to 0.93)	0.76 (0.67 to 0.85)	0.78 (0.69 to 0.89)
PH of stroke, n (%)	na	na	na	223 (1.27)	126 (0.59)	349 (0.90)
PH of IHD, n (%)	na	na	na	902 (4.96)	248 (1.16)	1150 (2.90)
LLAS	1.90±1.26	1.98±1.26	1.94±1.26	2.00±1.33	1.99±1.33	2.00±1.33

Where: CC-IMTmean, average of means of intima-media thickness in left and right common carotid arteries; CC-IMTmax, the highest value of maximum intima-media thickness in left and right common carotid arteries; CC-IMTmean-max, mean of maximum intima-media thickness in left and right common carotid arteries; P.H., personal history; IHD, ischemic heart disease; LLAS, lipid-lowering allelic score. Lipid-lowering medication (statins, fibrates, fish oil and/or resin in IMPROVE); and self-reported lipid-lowering medication in UKB). Binary variables are presented as n (%) and continuous variables are presented as mean ± SD or median (25%; 75%).

BMI, body mass index; CC-IMTmax, the highest value of maximum intima-media thickness in left and right common carotid arteries; CC-IMTmean, average of means of intima-media thickness in left and right common carotid arteries; CC-IMTmean-max, mean of maximum intima-media thickness in left and right common carotid arteries; HDL-C, high-density lipoprotein cholesterol; IHD, ischaemic heart disease; LDL-C, low-density lipoprotein cholesterol; LLAS, Lipid-Lowering Allelic Score; PCSK9, proprotein convertase subtilisin/kexin type 9; PH, personal history; UKB, UK Biobank.

### Associations of PCSK9 variants with lipids and PCSK9 levels in IMPROVE

The effects of the four variants on lipids levels and PCSK9 are shown in table 2. Of these variants, only rs11591147-T (ie, R46L) was significantly associated with LDL-C levels with the effect directions (ie, beta <0) in line with expectation (table 2). All variants showed consistent effect directions for PCSK9 levels and LDL-C levels. Sex-stratified results were also consistent, although not all associations reached statistical significance (p<0.0125).

**Table 2:** Association between single variants and lipids and PCSK9 measures in the IMPROVE Study

			All		Women		Men	
			(n=2892)		(n=1560)		(n=1332)	
	Variant	Allele	Beta±SE	P value	Beta±SE	P value	Beta±SE	P value
LDL-C	rs11206510	C	−0.041±0.032	0.199	−0.037±0.043	0.395	−0.053±0.046	0.248
	rs2479409	G	0.034±0.025	0.174	0.003±0.036	0.937	0.072±0.035	0.043
	rs11591147	T	−0.493±0.105	**2.97 × 10^–6^**	−0.576±0.165	**0.0005**	−0.426±0.134	**0.002**
	rs11583680	T	−0.006±0.037	0.877	0.022±0.050	0.654	−0.050±0.055	0.372
HDL-C	rs11206510	C	0.000±0.012	0.995	−0.005±0.017	0.758	0.004±0.015	0.782
	rs2479409	G	−0.007±0.009	0.481	−0.017±0.014	0.230	0.005±0.012	0.682
	rs11591147	T	−0.053±0.038	0.166	−0.072±0.063	0.255	−0.038±0.044	0.385
	rs11583680	T	−0.005±0.014	0.719	0.005±0.020	0.802	−0.019±0.018	0.291
Triglycerides	rs11206510	C	0.008±0.018	0.674	0.026±0.023	0.270	−0.009±0.029	0.743
	rs2479409	G	−0.005±0.015	0.736	−0.002±0.019	0.913	−0.010±0.022	0.654
	rs11591147	T	−0.045±0.060	0.455	0.032±0.086	0.706	−0.104±0.084	0.215
	rs11583680	T	0.023±0.021	0.293	0.01±0.027	0.698	0.047±0.035	0.181
PCSK9 (Model 1)	rs11206510	C	−0.042±0.011	**0.0001**	−0.040±0.014	**0.004**	−0.043±0.018	0.015
	rs2479409	G	0.039±0.009	**9.70 × 10^–6^**	0.030±0.011	**0.009**	0.047±0.014	**0.001**
	rs11591147	T	−0.452±0.035	**5.82 × 10^–37^**	−0.363±0.050	**8.71 × 10^–13^**	−0.521±0.049	**2.98 × 10^–25^**
	rs11583680	T	−0.054±0.013	**3.08 × 10^–5^**	−0.040±0.016	**0.0121**	−0.070±0.021	**0.001**
PCSK9 (Model 2)	rs11206510	C	−0.033±0.011	**0.002**	−0.030±0.013	0.025	−0.036±0.017	0.033
	rs2479409	G	0.035±0.008	**4.35 × 10^–5^**	0.027±0.011	0.014	0.041±0.013	**0.002**
	rs11591147	T	−0.422±0.034	**5.52 × 10^–35^**	−0.327±0.048	**1.85 × 10^–11^**	−0.496±0.047	**1.23 × 10^–24^**
	rs11583680	T	−0.049±0.012	**8.92 × 10^–5^**	−0.040±0.015	**0.008**	−0.058±0.020	**0.005**
PCSK9 (Model 3)	rs11206510	C	−0.043±0.016	**0.007**	−0.030±0.024	0.219	−0.048±0.021	0.020
	rs2479409	G	0.034±0.012	**0.004**	0.014±0.018	0.456	0.045±0.016	**0.005**
	rs11591147	T	−0.432±0.050	**1.25 × 10^–17^**	−0.332±0.104	**0.001**	−0.458±0.058	**1.12 × 10^–14^**
	rs11583680	T	−0.051±0.018	**0.006**	−0.008±0.028	0.788	−0.075±0.025	**0.002**

Where: rs11206510-C, rs11591147-T, rs11583680-T (all lipid lowering alleles) and rs2479409-G (lipid increasing allele). Lipid analyses were adjusted for sex, age, population structure (MDS1–3) and lipid-lowering medication; PCSK9 analyses were adjusted for: Model 1: adjusted for sex, age and population structure (MDS1-3); Model 2: as model 1 + use of lipid-lowering medication (including statins, fibrates, fish oil and/or resin); Model 3: as model 1 + PCSK9 independent determinants (use of statins and/or fibrates, total cholesterol, HDL-C, uric acid, personal history of hypertriglyceridaemia and/or hypercholesterolaemia, hip circumference, consumption of fish and/or wine, pack-years, family history of diabetes). p<0.0125 for statistical significance (Bonferroni correction for four comparisons) are highlighted in bold.

HDL-C, high-density lipoprotein cholesterol; LDL-C, low-density lipoprotein cholesterol; MDS1–3, Multidimensional scaling components 1, 2 and 3; PCSK9, proprotein convertase subtilisin/kexin type 9.

### Associations of PCSK9 variants with CC-IMT variables in IMPROVE

No significant associations were observed between individual variants and CC-IMTmean, CC-IMTmax or CC-IMTmean-max in sex-combined or sex-stratified analyses (online supplemental table S2). However, effect directions were consistent with LDL-C for CC-IMTmax and CC-IMTmean-max in sex-combined analyses, and for CC-IMTmax in women.

No significant associations were observed when analyses were stratified by lipid-lowering treatment (online supplemental table S3). In untreated subjects, the effect direction was in line with that expected for all variants.

### Associations of PCSK9 variants with CC-IMT variables (mean, max and mean-max) in UKB

A significant association was observed between rs11591147-T and CC-IMTmean, CC-IMTmax and CC-IMTmean-max in sex-combined analyses and in men (online supplemental table S4). The effect direction of these associations was consistent with that for LDL-C for CC-IMTmean and CC-IMTmean-max in sex-combined analyses, and for all CC-IMT variables in men.

Analyses stratified by lipid-lowering medication demonstrated significant associations between rs11591147-T and CC-IMT variables in all participants, and in untreated participants (online supplemental table S5). In contrast, rs2479409-G was only significantly associated with C-IMTmean in all untreated participants (online supplemental table S5).

### Associations of LLAS with CC-IMT in IMPROVE

The lipid-lowering LLAS (combined effects of four genetic variants) was significantly associated (p<0.05) with all CC-IMT variables in women but not in men or the sex-combined analyses (table 3). Beta values were in the expected direction (beta <0) for all CC-IMT variables in the sex-combined analyses and in women, that is, the higher the LLAS, the more lipid-lowering alleles, the lower the values of CC-IMTmean, CC-IMTmax and CC-IMTmean-max. In women-only analyses, the LLAS explained 0.3% of the variance for CC-IMTmean and CC-IMTmax (R^2^=0.003), and 0.4% of the variance for CC-IMTmean-max (R^2^=0.004) (table 3). The interaction between LLAS and sex for CC-IMTmax was borderline statistically significant (p=0.051).

**Table 3:** Associations of the LLAS with CC-IMT variables in IMPROVE and UKB

	Men			Women			All			Interaction
IMPROVE	(n=1332)			(n=1560)			(n=2892)			LLAS × sex
	Beta±SE	R^2^	*P value*	Beta±SE	R^2^	*P value*	Beta±SE	R^2^	*P value*	*P*_interaction_ value
CC-IMTmean	0.001±0.005	3E-05	0.846	−0.007±0.003	0.003	**0.035**	−0.003±0.003	0.0005	0.238	0.128
CC-IMTmax	0.004±0.007	0.0002	0.574	−0.013±0.006	0.004	**0.019**	−0.005±0.004	0.0005	0.248	**0.051**
CC-IMTmean-max	0.001±0.005	1E-05	0.897	−0.009±0.004	0.003	**0.030**	−0.004±0.003	0.0006	0.191	0.140
UKB	(n=19 619)			(n=20 893)			(n=40 512)			LLAS × sex
	Beta±SE	R^2^	*P value*	Beta±SE	R^2^	*P value*	Beta±SE	R^2^	*P value*	*P*_interaction_ value
CC-IMTmean	−0.003±0.001	0.14	**0.006**	−0.001±0.001	0.199	0.410	−0.002±0.001	0.193	**0.008**	0.099
CC-IMTmax	−0.004±0.001	0.096	**0.014**	−0.001±0.001	0.136	0.370	−0.002±0.001	0.145	**0.014**	0.205
CC-IMTmean-max	−0.003±0.001	0.131	**0.008**	−0.001±0.001	0.186	0.550	−0.002±0.001	0.187	**0.016**	0.097

Where: CC-IMTmean, average of means of intima-media thickness in left and right common carotid arteries; CC-IMTmax, the highest value of maximum intima-media thickness in left and right common carotid arteries; CC-IMTmean-max, mean of maximum intima-media thickness in left and right common carotid arteries. IMPROVE analyses were adjusted for (sex), age, population structure (MDS1-3), and use of lipid-lowering medication (statins, fibrates, fish oil and/or resin). UKB analyses were adjusted for (sex), age, population structure (PGC1-8), genotyping chip and self-reported use of lipid-lowering medication. p< 0.05 for statistical significance are highlighted in bold.

CC-IMTmax, the highest value of maximum intima-media thickness in left and right common carotid arteries; CC-IMTmean, average of means of intima-media thickness in left and right common carotid arteries; CC-IMTmean-max, mean of maximum intima-media thickness in left and right common carotid arteries; LLAS, Lipid-Lowering Allelic Score; MDS1–3, multidimensional scaling components 1, 2 and 3; PGC1–8, eight principal genetic components; UKB, UK Biobank.

Analysis stratified by lipid-lowering medication demonstrated no significant associations between the LLAS and CC-IMT variables (online supplemental table S6). However, beta values were in the expected direction (beta <0) for all CC-IMT variables in untreated subjects, and only in CC-IMTmean and CC-IMTmean-max in treated subjects. No significant LLAS by lipid-lowering medication interaction was observed (online supplemental table S6).

### Associations of LLAS with CC-IMT variables in UKB

Significant associations were observed between the LLAS and CC-IMT variables in the sex-combined analyses and in men but not women (table 3). In sex-combined analyses, the LLAS explained approximately 20% of the variance for CC-IMTmean (R^2^=0.193), CC-IMTmean-max (R^2^=0.187) and 14.5% for CC-IMTmax (R^2^=0.145). All beta values were in the expected direction (beta <0). No significant interaction was observed between LLAS and sex in UKB (table 3).

Analyses stratified by lipid-lowering medication in UKB (online supplemental table S6) demonstrated significant associations between LLAS and all IMT measures in untreated individuals, consistent with the findings observed in the entire group (online supplemental table S6). LLAS explained approximately 20% of the variation in CC-IMTmean and in CC-IMTmean-max, as well as about 14% of the variation in CC-IMTmax in untreated subjects (online supplemental table S6). No statistically significant interaction between LLAS and treatment was observed (online supplemental table S6).

### Combined effects of LLAS on CC-IMT variables in IMPROVE and UKB

Forest plots of LLAS effects demonstrated a consistent negative impact on all CC-IMT variables in IMPROVE, UKB and the meta-analyses (figure 1); however the effect was more convincing for UKB (n=40 512) and the meta-analyses (n=43 404) than for IMPROVE (n=2892).

### PCSK9 levels in the IMPROVE Study and association with LLAS

**Figure 2:**
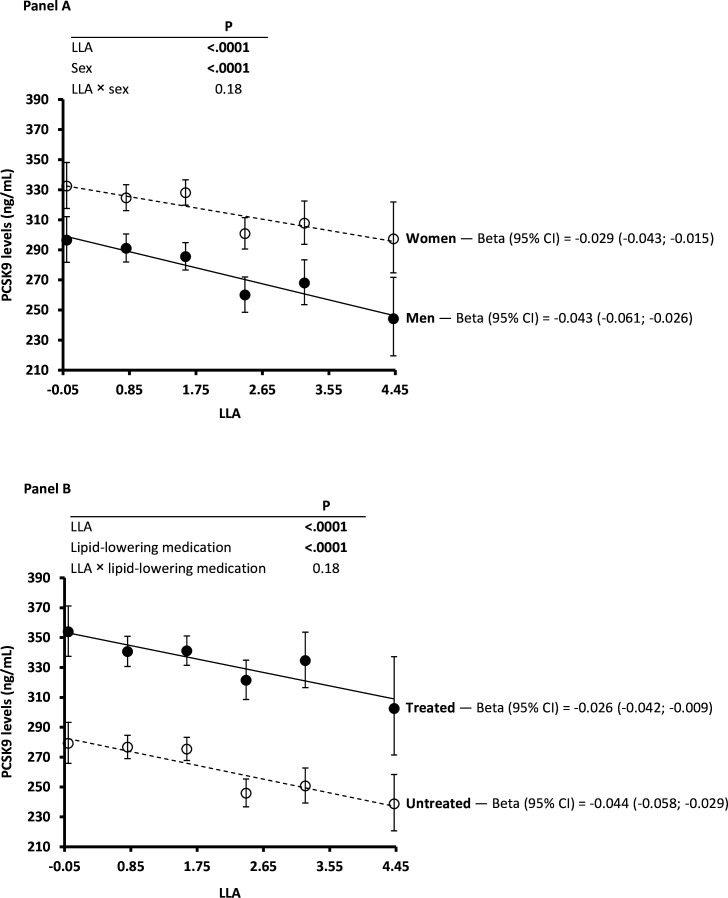
PCSK9 levels (adjusted geometric mean) versus lipid-lowering allele score (LLAS) after participants’ stratification according to sex (panel A) or use of lipid-lowering medication (panel B) in the IMPROVE Study. Beta (95% CI) refers to PCSK9 (natural log). The LLAS was standardised prior to analysis. In panel A, the analysis was adjusted for age, population structure (MDS1–3) and use of lipid-lowering medication (statins, fibrates, fish oil and/or resin). In panel B, the analysis was adjusted for sex, age and population structure (MDS1–3). The value of p_interaction_ was not statistically significant (ie, value of p_interaction_<0.05). LLAS, Lipid-Lowering Allelic Score; MDS1–3, multidimensional scaling components 1, 2 and 3; PCSK9, proprotein convertase subtilisin/kexin type 9.

Figure 2 shows the association between LLAS and (adjusted geometric mean) PCSK9 levels in men and women (panel A) as well as in treated and untreated individuals (panel B). These PCSK9 levels were significantly higher in women compared with men (p<0.0001) and in treated compared with untreated individuals (p<0.0001).

PCSK9 levels decreased with increasing LLAS values and with no difference between men and women (p_sex interaction_=0.18) or between treated and untreated individuals (p_lipid-lowering medication interaction_=0.18). PCSK9 levels were not available in UKB.

## Discussion

Our results demonstrated that LLAS including four *PCSK9* genetic variants was significantly associated with reduced atherosclerosis, assessed by CC-IMTs, in the high-CVD risk IMPROVE cohort (n=2892) and the general population UKB cohort (n=40 512). The effect was modest and did not differ significantly by sex or use of lipid-lowering medication.

Genetic variants in the *PCSK9* coding gene are among those with the greatest effect sizes. In particular, the R46L variant (rs11591147-T) has been associated with reduced PCSK9 levels,^29^ lower LDL-C levels^16^ and a lower prevalence and/or incidence of ischaemic heart disease.^30^ Most studies report a negative association between this variant and LDL-C levels (well described in ad hoc meta-analysis^31^), with one study reporting no association.^17^

We have demonstrated that the minor alleles of four *PCSK9* variants known to be associated with lower LDL-C levels (with MAFs of 2%–34%) were associated (although not all significantly) with circulating PCSK9 and LDL-C levels in IMPROVE. We also demonstrated that rs11591147-T (R46L) was associated with CC-IMT in UKB. The null effect in IMPROVE is likely due to the smaller sample size. We further demonstrated that LLAS was associated with CC-IMT measures in UKB, and when UKB and IMPROVE were meta-analysed. No lipid-lowering medication interaction was observed. The observed associations between *PCSK9* variants and CC-IMT underscore the role of LDL-C metabolism in arterial wall changes, supporting the potential utility of *PCSK9* genetic risk profiling in refining CVD risk prediction and early intervention strategies.

The choice of variants fulfilling the criteria set by Schmidt^18^ (online supplemental material), focusing on variants that influenced LDL-C levels, means that we didn’t assess other *PCSK9* variants previously associated with atherosclerosis.^15 16^ Of the variants previously associated, rs67608943 was not available on the genotyping platform used for IMPROVE and rs28362286 is a rare variant (MAF<1% in European ancestry (https://www.ncbi.nlm.nih.gov/snp/rs28362286/)). Rs562556 was not available in the GWAS catalog. Rs505151 has previously been associated with cholesterol levels only in trans-ancestry analyses^32 33^ or non-standard genome-wide association study (GWAS) methods in Europeans,^34^ but not in the Global Lipids Genetics Consortium meta-analysis^35^ (which was a criteria for variant inclusion).

Our finding that these genetic variants (individually and LLAS) influence PCSK9 levels, LDL-C levels and CC-IMT measures in a consistent fashion is in line with the known roles of PCSK9 on LDL-C levels and LDL-C on atherosclerotic burden. To our knowledge, this study is the first to demonstrate a significant, although modest, association between CC-IMT and a lipid-lowering *PCSK9* LLAS.

### Strengths and limitations

CC-IMT is widely used as a marker of subclinical atherosclerosis^2^ and is associated with CVD risk;^19^ however, it also reflects vascular remodelling, which may be influenced by factors beyond lipid accumulation.

Strengths of this study include: inclusion of two substantially different cohorts, IMPROVE (high CVD-risk, Pan-European recruitment) and the UKB (general population, UK recruitment), which enhances robustness and generalisability of the findings; use of *PCSK9* genetic variants with known functional effects on LDL-C levels; use of LLAS to assess combined genetic effects (as individual variants do not act in isolation).

Although differences in data collection protocols between the two cohorts prevented full control for potential effects of diet and nutritional status, the consistent findings from both IMPROVE and UKB—despite their distinct dietary contexts—suggest that significant residual confounding by diet is unlikely. Similarly, methodological and precision-related differences in CC-IMT measurement between IMPROVE and UKB could, in principle, have influenced the results. However, the consistency of effect sizes across cohorts and the alignment of SEs with respective population sizes suggest that such differences are unlikely to have introduced substantial bias into the pooled analysis.

There are also limitations: individual variants did not consistently demonstrate robust effects on CC-IMT; the possibility of unmeasured factors—such as dietary habits, physical activity or other genetic influences—influencing the associations between *PCSK9* variants and CC-IMT remains; UKB has a well-recognised healthy bias^36^ and relied on self-report of medication. Overall, it is likely that biases in the data would result in underestimation (not overestimation) of effects. Finally, the absence of heterogeneity observed in the fixed-effects meta-analysis may have been influenced by the low number of studies (just two) compared. Our analyses focused on only variants in the *PCSK9* gene, which have modest effects on PCSK9 and LDL-C levels as well as CC-IMT measures. We did not consider or adjust for genetic variants in other genes, some of which have bigger effects on LDL-C (but potentially no effect on PCSK9), as this would prevent the assessment of the genetic variant effects on the mechanism of altered PCSK9 to altered LDL-C to altered CC-IMT.

### Conclusion

In conclusion, we have demonstrated genetic variants in *PCSK9* which influence PCSK9 and LDL-C levels, also influence CC-IMT, and are likely to be part of a causal pathway for atherosclerosis. Genetic risk profiling, including these variants, could be useful in future precision medicine strategies, where individuals most at risk can be targeted for intervention prior to overt disease development.

## Supplementary material

10.1136/openhrt-2025-003348 online supplemental file 1

10.1136/openhrt-2025-003348 online supplemental file 2

10.1136/openhrt-2025-003348 online supplemental file 3

10.1136/openhrt-2025-003348 online supplemental file 4

10.1136/openhrt-2025-003348 online supplemental file 5

10.1136/openhrt-2025-003348 online supplemental file 6

10.1136/openhrt-2025-003348 online supplemental file 7

## Data Availability

Data are available upon reasonable request.
