# Supplementary material for: PCSK9 genetic variants, carotid atherosclerosis and vascular remodelling
Source: Open Heart. 2025 Oct 10;12(2):e003348. doi: 10.1136/openhrt-2025-003348 (PMC12517018; doi:10.1136/openhrt-2025-003348)
Supplement: online supplemental file 7 [file openhrt-12-2-s007.docx]

# **SUPPLEMENTAL MATERIAL**

## **The IMPROVE study group**

***Centro Cardiologico Monzino, IRCCS, Milan, Italy****: Beatrice Frigerio, Daniela Sansaro, Alessio Ravani, Daniela Coggi, Alice Bonomi, Nicolò Capra, Mauro Amato, Damiano Baldassarre.*

***Department of Medical Biotechnology and Translational Medicine, Università degli Studi di Milano, Milan, Italy****: Damiano Baldassarre.*

***Maria Cecilia Hospital, Cotignola (RA), Italy****: Fabrizio Veglia, Elena Tremoli.*

***Dipartimento di Scienze Farmacologiche e Biomolecolari, Università degli Studi di Milano, Milan, Italy****: Laura Calabresi, Cesare R. Sirtori.*

***Department of Medicine Solna, Division of Cardiovascular Medicine, Karolinska Institutet, Stockholm, Sweden****: Per Eriksson, Rona J. Strawbridge, Bruna Gigante, Angela Silveira, Anders Hamsten.*

***Karolinska University Hospital, Solna, Stockholm, Sweden****: Angela Silveira, Per Eriksson, Anders Hamsten.*

***Institute of Health and Wellbeing, University of Glasgow, Glasgow, United Kingdom****:* *Rona J. Strawbridge.*

***Health Data Research UK****: Rona J. Strawbridge.*

***Division of Cardiovascular and Nutritional Epidemiology, Institute of Environmental Medicine, Karolinska Institutet****: Karin Leander, Federica Laguzzi, Ulf de Faire.*

***Cardiovascular Genetics, Institute Cardiovascular Science, University College of London, Rayne Building, University Street, London, United Kingdom****: Steve E. Humphries, Jackie A. Cooper, Jayshree Acharya.*

***Foundation for Research in Health Exercise and Nutrition, Kuopio Research Institute of Exercise Medicine, Kuopio, Finland****: Kai Savonen, Kirsi Huttunen, Eva Rauramaa, Ilkka M. Penttila, Jukka Törrönen.*

***Department of Clinical Physiology and Nuclear Medicine, Kuopio University Hospital, Kuopio, Finland****: Kai Savonen.*

***Department of Medicine, University Medical Center Groningen, Groningen & Isala Clinics Zwolle, Department of Medicine; the Netherlands****: Andries J. Smit, A.I. van Gessel, A.M van Roon, A. Nicolai, D.J. Mulder, G.H. Smeets.*

***Assistance Publique - Hopitaux de Paris; Service Endocrinologie-Metabolisme, Groupe Hôspitalier Pitié-Salpetriere, Unités de Prévention Cardiovasculaire, Paris, France****:* *Philippe Giral, Anatole Kontush, Alain Carrié, Antonio Gallo.*

***Institute of Public Health and Clinical Nutrition, University of Eastern Finland, Kuopio Campus****:*

*Sudhir Kurl, J. Karppi, T. Nurmi, K. Nyyssönen, T.P. Tuomainen, J.Tuomainen, J. Kauhanen.*

***Internal Medicine, Angiology and Arteriosclerosis Diseases, Department of Medicine and Sugery, University of Perugia, Perugia, Italy****: Matteo Pirro, M.R. Mannarino, G. Vaudo, V. Bianconi, E. Marini, F. Figorilli.*

## ***Supplemental Methods***

***Selection of genetic variants***

Genetic variants were identified as per the definition used by Schmidt et al.^1^ namely: (1) a robust association with LDL-C (as observed by the Global Lipids Genetics Consortium^2^), (2) a low pairwise linkage disequilibrium (LD) (r^2^≤0.30) with other variants in that region (using 1000 Genomes CEU data); and (3) a combined annotation dependent depletion (CADD) score^3^ that evaluates potential functionality.

## ***Study design***

The IMPROVE study was approved by 6 independent ethics committees, i.e.: the Regional Ethics Review Board at Karolinska Institutet, Stockholm, Sweden (approval ID: Dnr 2003/03-115, 17 February 2003); the Institutional Review Board of the Health Department of the Hospital “Ospedale Niguarda Ca’ Granda”, Milan, Italy (approval ID: 2042/03, 3 June 2003); the Medical Ethics Review Committee, Academic Hospital Groningen, Groningen, the Netherlands (approval ID: METc 2003/054, 12 May 2003); the Ethics Committee of the Umbrian Health Authorities, Perugia, Italy (approval ID: N 2725/03/A, 6 February 2003); the Consultative Committee for the Protection of Persons in Biomedical Research (CCPPRB) at Hôspital Pitie Salpétrière, Paris, France (approval ID: CCP61-03, 2003); the Research Ethics Committee of the University of Kuopio and Kuopio University Hospital, Kuopio, Finland (approval ID: 140/2002, 10 October 2002).

The Research Ethics Committee of the University of Kuopio and Kuopio University Hospital, Kuopio, Finland (approval ID: 39/2003, 11 February 2003) has withdrawn the permission to use its data as of January 2023. Therefore, such data have been not used in this article.

## ***IMPROVE common carotid (CC) ultrasonographic variables measurements***

Ultrasonographic scans were performed by using seven identical devices (Technos system - Esaote, Genoa, Italy) equipped with a 5-10-MHz linear array probe. Machines were calibrated all with a phantom at baseline and checked 1 year later. The far walls of left and right common carotid (CC) arteries, bifurcations (Bif), and internal carotid arteries (ICA) were visualized in 3 different scan angles (lateral, anterior, and posterior) and recorded on sVHS videotapes. In this study, we considered CC ultrasonographic variables measured in two scan angles (lateral and anterior) as these were deemed most comparable to the scan angles used by UKB. The CC intima-media thickness (CC-IMT) was measured, at least, in 3 different frames. Carotid plaques (defined as maximum IMT > 1.5 mm) were incorporated in IMT measurements. CC ultrasonographic variables considered were: CC-IMTmean, i.e. the average of means of IMT in left and right CC arteries; CC-IMTmax, i.e. the highest value of maximum IMT in left and right CC arteries; CC-IMTmean-max, i.e. the mean of maximum IMT in left and right CC arteries. All the measurements were made in the Italian centre of Milan by using a dedicated software (M’Ath, Metris SRL, Argenteuil, France)^4^.

## ***UK Biobank (UKB) CC ultrasonographic variables measurements***

Full details of the carotid IMT (CC-IMT) examination protocol are available at <https://biobank.ctsu.ox.ac.uk/crystal/label.cgi?id=101>, have been described in detail ^5^and analyses of CC-IMT measurements has previously been described^6^. In summary, a pilot phase of CC-IMT phenotyping began in 2015, with N=2,272 individuals being imaged at 18 centres (of which 8 centres accounted for 98% of the sample) and extensive manual quality control being conducted. After review of quality control data, all centres recruited and recorded automated measurements (10 centres accounted for 93% of the sample). In all cases, ultrasound was used to measure the far wall of the distal CC artery on the left and right (two angles on each side) with automated software recording images and values of mean and maximum intima-media (UKB data fields 22670-22681). Recruitment for the imaging visit continues. To date, N=49,088 individuals have ultrasound measurements of the CC-IMT, and are unrelated individuals of white British ancestry.

From raw data we calculated: CC-IMTmean, average of 4 mean measures (2 for each of the left and right CC arteries); CC-IMTmax, maximum IMT, where the largest of the 4 maximum IMT measures was used; CC-IMTmean-max, mean of 4 maximum measures (2 for each of the left and right CC arteries). CC-IMT measures were reported in mm. Where >1 value was missing due to poor quality of the image, the participant was excluded from analyses. Self-reported use of lipid-lowering medication (field 6153) and self-report of a diagnosis of stroke or ischemic heart disease (IHD) (including heart attack and angina) (field 6150) were used.

## ***IMPROVE PCSK9 measurements***

Plasma was isolated from blood samples and diluted 1:20 before incubation on microplates pre-coated with a human PCSK9-specific monoclonal antibody^7^. PCSK9 concentrations in the samples were obtained by generating a four-parameter logistic curve-fit. The lowest detectable PCSK9 level was 0.219 ng/mL. Intra- and inter-assay coefficients of variation were 5.4 ± 1.2% and 4.8 ± 1.0%, respectively^7^.

## ***Body mass index (BMI) and waist/hip ratio measurements in the IMPROVE and UKB studies***

BMI, an index of obesity and healthy weight^8^, was calculated by dividing the subject weight, expressed in kg, by the square of the subject height, expressed in m. Therefore, the BMI value is expressed in Kg/m^2^.

Waist/hip ratio is considered an index of fat mass distribution in the body^9^. It was calculated by dividing the waist circumference, expressed in cm, by the hip circumference, expressed in cm.

## ***IMPROVE lipid measurements***

Venous blood samples were collected after an overnight fasting and maintained at 4°C until the plasma was separated, aliquoted, and stored at -80°C. Serum concentrations of high-density lipoprotein cholesterol (HDL-C) and triglycerides were analysed in a centralized laboratory by enzymatic methods^10, 11^ and presented in mmol/L. LDL-C levels were calculated with Friedewald’s formula^12^.

**References**

[1] Schmidt, AF, Holmes, MV, Preiss, D, et al., Phenome-wide association analysis of LDL-cholesterol lowering genetic variants in PCSK9, BMC Cardiovasc Disord, 2019;19:240.

[2] Willer, CJ, Sanna, S, Jackson, AU, et al., Newly identified loci that influence lipid concentrations and risk of coronary artery disease, Nat Genet, 2008;40:161-169.

[3] Kircher, M, Witten, DM, Jain, P, et al., A general framework for estimating the relative pathogenicity of human genetic variants, Nat Genet, 2014;46:310-315.

[4] Beux, F, Carmassi, S, Salvetti, MV, et al., Automatic evaluation of arterial diameter variation from vascular echographic images, Ultrasound Med Biol, 2001;27:1621-1629.

[5] Coffey, S, Lewandowski, AJ, Garratt, S, et al., Protocol and quality assurance for carotid imaging in 100,000 participants of UK Biobank: development and assessment, Eur J Prev Cardiol, 2017;24:1799-1806.

[6] Strawbridge, RJ, Ward, J, Bailey, MES, et al., Carotid Intima-Media Thickness: Novel Loci, Sex-Specific Effects, and Genetic Correlations With Obesity and Glucometabolic Traits in UK Biobank, Arterioscler Thromb Vasc Biol, 2020;40:446-461.

[7] Ferri, N, Ruscica, M, Coggi, D, et al., Sex-specific predictors of PCSK9 levels in a European population: The IMPROVE study, Atherosclerosis, 2020;309:39-46.

[8] Gutin, I, In BMI We Trust: Reframing the Body Mass Index as a Measure of Health, Soc Theory Health, 2018;16:256-271.

[9] Min, YI, Gao, Y, Anugu, P, et al., Obesity and overall mortality: findings from the Jackson Heart Study, BMC Public Health, 2021;21:50.

[10] Bucolo, G and David, H, Quantitative determination of serum triglycerides by the use of enzymes, Clin Chem, 1973;19:476-482.

[11] Warnick, GR, Benderson, J and Albers, JJ, Dextran sulfate-Mg2+ precipitation procedure for quantitation of high-density-lipoprotein cholesterol, Clin Chem, 1982;28:1379-1388.

[12] Friedewald, WT, Levy, RI and Fredrickson, DS, Estimation of the concentration of low-density lipoprotein cholesterol in plasma, without use of the preparative ultracentrifuge, Clin Chem, 1972;18:499-502.

## **Supplemental Figure**

### **S Figure 1**

| 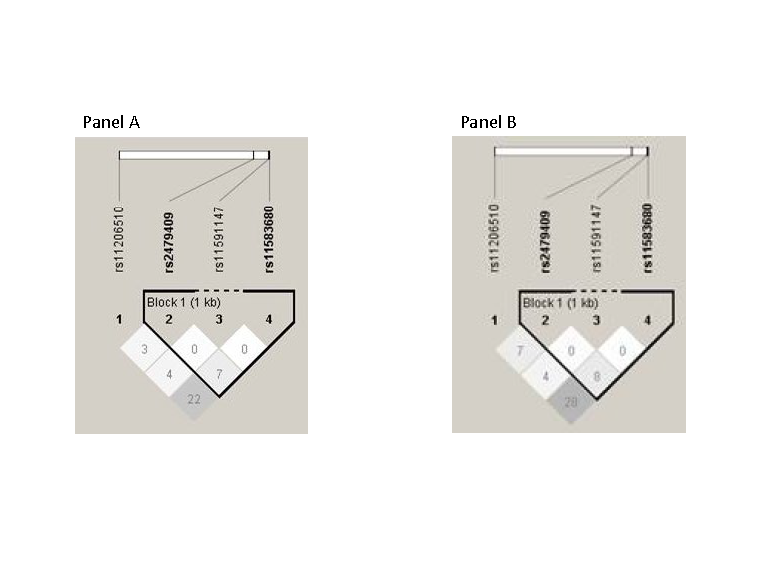 |
| --- |
| Linkage disequilibrium (LD) between the four variants included in the analyses for the IMPROVE study (Panel A) and the UKB study (Panel B). Colours and values reflect LD R^2^. Of note, the Block indication is an artifact of the software and is not accurate to the LD measure used. |

**Supplemental Tables**

S Table 1: Minor allele frequency (MAF) and Hardy-Weinberg Equilibrium (HWE) p-value of PCSK9 variants in the IMPROVE and UKB studies.

S Table 2. Associations between single variants and CC-IMT variables in IMPROVE (sex-combined and sex-stratified analyses)

S Table 3. Associations between single variants and CC-IMT variables in IMPROVE (treatment-combined and treatment-stratified analyses)

S Table 4. Associations between single variants and CC-IMT variables in UKB (sex-combined and sex-stratified analyses)

S Table 5. Associations between single variants and CC-IMT variables in UKB (treatment-combined and treatment-stratified analyses)

S Table 6. Association between PRS and CC-IMTmean, CC-IMTmax and CC-IMTmean-max in the IMPROVE study, stratified by use of lipid-lowering medication, and interaction between PRS and use of lipid-lowering medication.
